# Is bioelectrical impedance accurate for use in large epidemiological studies?

**DOI:** 10.1186/1475-2891-7-26

**Published:** 2008-09-09

**Authors:** Mahshid Dehghan, Anwar T Merchant

**Affiliations:** 1 Population Health Research Institute, McMaster University, Hamilton, ON, Canada; 2 Department of Medicine, McMaster University, Hamilton, ON, Canada; 3 Department of Clinical Epidemiology and Biostatistics, and Population Health Research Institute, McMaster University, Hamilton, ON, Canada

## Abstract

Percentage of body fat is strongly associated with the risk of several chronic diseases but its accurate measurement is difficult. Bioelectrical impedance analysis (BIA) is a relatively simple, quick and non-invasive technique, to measure body composition. It measures body fat accurately in controlled clinical conditions but its performance in the field is inconsistent. In large epidemiologic studies simpler surrogate techniques such as body mass index (BMI), waist circumference, and waist-hip ratio are frequently used instead of BIA to measure body fatness. We reviewed the rationale, theory, and technique of recently developed systems such as foot (or hand)-to-foot BIA measurement, and the elements that could influence its results in large epidemiologic studies. BIA results are influenced by factors such as the environment, ethnicity, phase of menstrual cycle, and underlying medical conditions. We concluded that BIA measurements validated for specific ethnic groups, populations and conditions can accurately measure body fat in those populations, but not others and suggest that for large epdiemiological studies with diverse populations BIA may not be the appropriate choice for body composition measurement unless specific calibration equations are developed for different groups participating in the study.

## Introduction

In this review we discuss the issues associated with the application of bioelectrical impedance analysis (BIA) to measure body composition in large epidemiologic studies with multiethnic populations. The review is limited to healthy adults and does not include children, adolescents, elderly, and unhealthy individuals. The most recent system such as foot (or hand) to foot system is the main focus of this review and the early tetra-polar electrode system will not be discussed. These recent models are readily available and easy to use.

Percent body fat is strongly associated with the risk of chronic diseases such as hypertension, dyslipidemia, diabetes mellitus, and coronary heart disease [1-4]. In epidemiological studies, surrogate measures of body fatness such as body mass index (BMI), waist circumference, waist-hip ratio and skin fold thickness have been used extensively. However, these techniques do not precisely characterize persons by body composition (percentage of body fat or muscle mass), and there is substantial variation across age, sex and ethnic groups [5-7]. Several techniques have been used to assess percent body fat in controlled laboratory conditions. These include underwater weighing (densitometry), dual energy x-ray absorptiometry (DEXA), bioelectrical impedance analysis (BIA) and magnetic resonance imaging (MRI). However, densitometry, DEXA, and MRI are expensive, inconvenient for the participant, and not feasible to conduct in the field because they require large specialized equipment. For these reasons, their use in large epidemiological studies is limited.

BIA, by contrast, is relatively simple, quick (takes only a few minutes), and non-invasive which gives reliable measurements of body composition with minimal intra- and inter-observer variability [8]; the results are available immediately and reproducible with <1% error on repeated measurements [9]. This technique became commercially available for the first time in the mid- 1980s [10], and requires inexpensive, portable equipment, making it an appealing alternative to assess body composition in epidemiological studies [11].

### Principles of bioelectrical impedance technique

BIA analysis is based on the principle that electric current flows at different rates through the body depending upon its composition. The body is composed mostly of water with ions, through which an electric current can flow. The water in the body is localized in two compartments: extra-cellular water (ECW, approximately 45%) and intracellular water (ICW, approximately 55%) [12]. On the other hand, the body also contains non-conducting materials (body fat) that provide resistance to the flow of electric current. Adipose tissue is significantly less conductive than muscle or bone [13]. The principal of BIA is that electric current passes through the body at a differential rate depending on body composition. Hence, there is a direct relationship between the concentrations of ions and the electrical *conductivity *and an indirect relationship exists between the ion concentration and the *resistance *of the solution.

**Body impedance (Z) **is defined as the opposition of a conductor to the flow of an alternating current, and consists of two components: resistance (R) and reactance (Xc). **Resistance (R) **is the major opposition of the conductor and at usual low frequency (50 kHz), the extra-cellular part of non-adipose tissue works as a resistor [14]. **Reactance **is an additional opposition or the storage of an electrical charge by a condenser for a short period of time; the lipid component of the membranes of the Body Cell Mass (BCM) behave as capacitors and reduce the flow of intracellular ions. In practice, impedance is the amount of dropped voltage when a small constant current (800 uA) with a fixed frequency (50 kHz) passes between electrodes spanning the body. However, lean tissue, which is rich in water and electrolytes, has minimal impedance and increases to a maximum when all lean tissue is replaced by fat/adipose tissue. Hence, lean body mass and Fat Mass (FM) can be calculated from the difference in conductivity [15].

The other assumptions for BIA measurement are that the body is a cylindrical-shaped ionic conductor with homogeneous composition, a fixed cross-sectional area and a uniform distribution of current density [16,17]; BIA measures the impedance to the flow of an electric current through the total body fluid. Therefore, the conductive volume (V) which represents total body water (TBW) or FFM is directly related to the square length of conductor (S) and inversely correlated to resistance of the cross-section area (R), while *p *is the specific receptivity of the conductor, yielding the equation: V = *p *× S^2^/R. Based on this assumption, the same arms and legs respectively contribute to almost 47% and 50% of whole body resistance despite contributing to 4% and 17% of body weight respectively. In contrast, the trunk, which contains 50% of the body mass, contributes only 5–12% of whole body resistance [12].

### Predictive equations

Many empirical equations have been developed for estimation of TBW, FFM and body cell mass (BCM), by using sex, age, weight, height and race as explanatory variables. However, predictive equations are generally population-specific and can be useful only for those populations with characteristics similar to those of the reference populations [18,19]. When these equations have been used to predict body composition in different populations, the results have been inconsistent. The developed predictive equations cannot be generalized to diverse populations. Heyward and Wagner reviewed the reliability and validity of different equations for African Americans, Asians and Indian Americans. They found that the majority of studies indicated that the BIA method is not accurate when a generalized equation is applied for different ethnic groups [20].

### Summary of bio-impedance technique

▪ Based on the principle that body fat impedes electric current more than body protein

▪ Impedance is a drop in voltage when a small constant current with a fixed frequency passes between electrodes spanning the body

▪ Predictive equations estimate TBW, FFM and body cell mass (BCM) using sex, age, weight, height and race

### Validity of BIA measurements

The human body is not uniform either in length, cross-sectional area, or ionic composition and this affects the accuracy of BIA measurements [15]. In addition, body impedance varies among different ethnic groups and influences the accuracy of BIA [21]. Validity of hand to hand (Omron BF306 BIA) with a 4-C model was tested among Chinese and Japanese participants which showed different levels of biases in predicted levels of body fat (SEE = 4.5% BF) which may have resulted from different levels of body fat, age and relative arm span [22]. Demura et al. in a sample of 50 Japanese men aged 18 to 27 y. validated foot-to-foot (Tanita, TBF-102), and hand-to-hand (Omron, HBF-300) and hand-to-foot (Selco, SIF-891) BIA analyzers against hydro-densitometry (HD) [23]. They found higher correlation between hand to foot (r = 0.96) than foot to foot (r = 0.71) against HD as a reference method and there was 2.2% to 3.3% overestimation when they used the manufacturer's equations, therefore, they developed new equations for their sample. Jebb et al. tested the validity of foot-to-foot (Tanita -350) among 104 men and 101 women recruited from Dunn Nutrition Centre using DEXA as a reference method. The observed limit of agreement for fat mass was ± 7.9 kg [24]. A number of other factors that influence BIA results are described in this section.

### Consumption of food or beverages

Although food or fluid intake before BIA measurement affects TBW and ECW, a general agreement on the ideal amount of time between food and fluid intake and BIA measurements has yet to be consolidated. It has been suggested that due to the large cross-sectional surface of the trunk, even fluid intake of up to 2 L is shown to be "electrically silent" during the first hour after consumption [25,26]. Kaminsky and Whaley (1993) compared body fat percentage measurements after 3 hours and 12 hours of fasting and found no significant difference between these values [27]. Lukaski et al., (1986) emphasizes that dehydration increases resistance by nearly 40 Ω, which results in a 5.0 kg underestimation of FFM [28] and Evans et al., (1998) showed increased impedance one hour after eating a heavy meal [25]. In contrast, investigators have reported that food intake, its absorption and the resulting increase in movement of fluid into the bloodstream from 2–4 hours before BIA measurement, decreases the impedance value from 4 to 15 Ω, or <3% and results in overestimation of FFM by almost 1.5 kg [29]. Slinde and Rossander-Hulthen, after giving standard food to 18 healthy subjects, measured BIA 18 times during 24 hr. Their results showed that percentage of body fat varied by 8.8% and 9.9% from the highest to the lowest measurement in women and men respectively [30]. In contrast, Chumlea et al., (1987) found no effect of food consumption before BIA measurement on impedance measurements [31]. For these reasons undertaking an overnight fast is recommended as a routine standardization technique before impedance measurements [17,32].

#### Exercise

Although exercise of mild intensity may not affect BIA measurements, moderate and intensive exercise before measurements may change the measured impedance by different mechanisms [33]. For example, exercise increases cardiac output and vascular perfusion and subsequently increases blood flow to skeletal muscle, which warms the muscle and decreases muscle resistance which results in reduced impedance [26]. In addition, intensive activity causes vasodilatation, an increase in skin temperature, which also reduces measured impedance [34]. Jogging or cycling at moderate intensities for 90–120 min decreases measured impedance by 50 to 70 Ω, which results in nearly a 12 kg overestimation of FFM [35]. Therefore, to reduce measurement error, BIA should not be performed within several hours of moderate to intensive exercise. In addition, the chosen mode for each individual may affect the accuracy of measurement. Swartz et al, in a well designed study, compared the % BF measured among high or moderately active and inactive individuals by hydrostatic weight and BIA using different athletic and adult modes in a foot-to-foot BIA (Tanita TBF-305). Their results showed that although the electrical impedance was not significantly different, the chosen adult mode for highly and moderately active individuals significantly overestimated the percent of body fat [36].

#### Medical conditions

Although some investigators have applied BIA method in various patients and clinical settings, it should be noted that there are some medical conditions which change serum electrolytes, hematocrit and blood flow, affecting Z and *p*, independent of body fluid volume [26]. Conversely, there are some other medical conditions, which via a change in fluid distribution alter Z measurements. Significant alteration in body hydration, fluid distribution and differences in the ratio of ECW to ICW caused by a medical condition will affect impedance measurements [37,38]. Among those conditions, the most significant confounding variable is edema of the distal extremities, which is mainly caused by peripheral venous insufficiency. This insufficiency may result from congestive heart failure, cirrhosis, nephrotic syndrome, hypoalbuminemia, and lympheodema [39]. Other medical conditions, which affect BIA validity, include cutaneous disease that may alter electrode-skin electrical transmission in patients with amputations, poliomyelitis and muscular dystrophies. These conditions will have significant effects on the application of BIA in the clinical population [17,40].

#### Environmental factors

Although environmental changes do not significantly affect actual whole body volume, they appear to alter the Z measurements by changing skin temperature. The result of several studies showed an inverse relation between skin temperature and impedance which means impedance increases with a lowering in temperature and decrease with a rise in skin temperature. Gudivaka et al observed 8% change in resistance at 50 kHz with 8.4°C change in skin temperature [41]. Thus, changes in cutaneous and muscle blood flow may have a large impact on BIA measurements in both clinical and field settings.

#### Within-subject variability

Due to increased progesterone plasma levels after ovulation and the change in hydration status, within-subject variability of impedance may be higher in women. The effect of this variability has been examined by several studies and various results have been reported. Gualdi-Russo et al., did not find significant differences in TBW estimated at different points in time during the follicular and premenstrual stages [42]. On the other hand, Gleichauf et al., suggested that the average of several measurements during a menstrual cycle could be considered as an estimation of body composition [43]. However, it has been recommended that BIA measurement not be taken at a time while the participant is experiencing large weight gain related to the menstrual cycle [44]. Menopause changes body composition and fat distribution and women experience a loss in lean mass and an increase in weight, fat mass and central fat deposition [45-49]. The ratio of fat/lean mass, especially in the lower part of the body increases [50,51], which may affect the estimated impedance as the current passes through the legs. Therefore, the accuracy of BIA measurements increases by applying specific prediction equations for postmenopausal women [52].

#### Ethnicity

In recent years, BIA has been extensively applied among different age groups of both sexes, including mostly Caucasian populations of USA and Europe, and several prediction equations have been developed for these samples [53-55]. Also, a few prediction equations have been developed based on samples from African Americans, Hispanics and Native Americans [56]. Stolarczky et al., (1997) showed that by applying population-specific equations for estimation of lean body mass among Native American women, the standard error for estimating (SEE) decreased from 8.1 kg to 2.6 kg [56]. However, it has been suggested that biological and physiological assumptions for estimation of body composition, which are mainly based on Caucasian samples, may not be accurate for other ethnic groups. **Hence, the validity of these equations must be tested in the population under study**. There are several factors responsible for ethnic differences, which may affect the extent and direction of the error while measuring body composition by BIA such as:

• **Fat distribution **Ethnicity affects fat patterning and consequently influences the validity of equations. It has been shown that the proportion of fat deposition on trunk varies by 5.7% between different ethnic groups of Asians, Mexican Americans, Caucasians and African Americans [57].

• **Body density **Body density may have a significant impact on the accuracy of estimated lean body mass and fat-free mass. Several studies showed that African Americans have greater body density and greater body mass cell compared to Caucasian Americans [58,59]. Swinburn et al., (1999) found that Polynesians in New Zealand have higher levels of fat-free mass and less body fat than Europeans at any given body mass index [60]. In contrast, Kyle et al., (2001) indicated that Japanese men and women had 10–12% higher body fat than Swiss men and women [55]. It has also been reported that Asian populations (Chinese, Malay, Singaporean Indians) have higher body fat percentages at a given BMI and Wang et al. reported a lower hydration of the FFM in Asians [6,61].

In prediction equation calculations, it has been assumed that the fat free mass density does not vary among different ethnic groups. Because the density of FFM differs between different ethnic groups, this assumption may be a major source of error.

• **Differences in proportional limb lengths **as mentioned before, impedance demonstrates a direct relationship between conductive volume (V) and the square length of a conductor (S). Since whole body impedance is mainly based on the impedance of limbs [62], the differences among different racial groups may mostly relate to differences in proportion of limb lengths [63]. This hypothesis is supported by several studies, for example, whole-body impedance of Nigerians was significantly greater than that of matched Caucasian individuals, but was not different among different tribes of Nigeria [11]. Also, several other studies showed that black populations have longer limbs than white populations and increased lumbar lordosis [64-66].

Generally speaking, based on the preceding hypothesis, regarding age, race, level of activity etc. it has been suggested that the general prediction equation across different age and ethnic groups should not be applied without cross validating the study population [61,67].

### Summary of factors impacting BIA results

▪ Contact between limbs and trunk

▪ Inaccurate body weight

▪ Consumption of food and drink (overnight fast suggested)

▪ Moderate to intense level physical activity 2–3 hours before measurement

▪ Medical conditions impacting fluid and electrolyte balance

▪ Ambient temperature (cold increases impedance)

▪ Individual characteristics (abdominal obesity, muscle mass, weight loss, menstrual cycle, menopause)

▪ Ethnic variation, possibly mediated by body density and proportional limb length

## Conclusion

BIA has become a popular method for estimation of body composition during the last two decades. Since 1990, more than 1600 published articles have been reported using BIA as a tool of body composition measurement [17,40,68] and our search with the key words of body composition and bioelectrical impedance showed that 235 articles were published in English between 2004 and 2006 and we found different levels of agreements between different BIA models and reference methods. Also, there are many different equations for BIA calibration thus results of studies should be compared with more caution. BIA seems to reasonably estimate body composition in controlled conditions for healthy and euvolemic adults by applying a population specific predictive equation and it is not recommended to generalize a few equations for international epidemiologic studies, which involve participants from diverse populations. As far as we know, for some ethnic groups such as South Asians or Middle Easterners, or African residing in Africa predictive equations have not yet been developed. Hence, it is necessary to develop new predictive equations or cross validate existing equations on new populations to be studied.

If the BIA equation is not appropriately chosen based on age, gender, level of physical activity, level of body fat and ethnicity, the results of the study will not be reliable.

Overall BIA is a useful tool for clinical studies, but for large epidemiological studies with diverse population, particularly in developing nations, BIA has limited use unless valuation studies are conducted specifically for the populations under study.

## Competing interests

The authors declare that they have no competing interests.

## Authors' contributions

MD ran the electronic searches, reviewed all abstracts and articles, coordinated and drafted the manuscript. ATM participated in reviewing the articles and helped to draft the manuscripts.
